# The Diagnosis and Management of Patients with Renal Colic across a Sample of US Hospitals: High CT Utilization Despite Low Rates of Admission and Inpatient Urologic Intervention

**DOI:** 10.1371/journal.pone.0169160

**Published:** 2017-01-03

**Authors:** Elizabeth M. Schoenfeld, Penelope S. Pekow, Meng-Shiou Shieh, Charles D. Scales, Tara Lagu, Peter K. Lindenauer

**Affiliations:** 1 Department of Emergency Medicine, Baystate Medical Center, Springfield, Massachusetts, United States of America; 2 Tufts University School of Medicine, Boston, Massachusetts, United States of America; 3 Center for Quality of Care Research, Baystate Medical Center, Springfield, Massachusetts, United States of America; 4 Duke Clinical Research Institute and Division of Urologic Surgery, Duke University School of Medicine, Durham, North Carolina, United States of America; 5 Department of Internal Medicine, Baystate Medical Center, Springfield, Massachusetts, United States of America; Istituto Di Ricerche Farmacologiche Mario Negri, ITALY

## Abstract

**Objectives:**

Symptomatic ureterolithiasis (renal colic) is a common Emergency Department (ED) complaint. Variation in practice surrounding the diagnosis and management of suspected renal colic could have substantial implications for both quality and cost of care as well as patient radiation burden. Previous literature has suggested that CT scanning has increased with no improvements in outcome, owing at least partially to the spontaneous passage of kidney stones in the majority of patients. Concerns about the rising medical radiation burden in the US necessitate scrutiny of current practices and viable alternatives. Our objective was to use data from a diverse sample of US EDs to examine rates of and variation in the use of CT scanning, admission, and inpatient procedures for patients with renal colic and analyze the influence of patient and hospital factors on the diagnostic testing and treatment patterns for patients with suspected renal colic.

**Methods:**

We conducted a retrospective cohort study of adult patients who received a diagnosis of renal colic via a visit to an ED at 444 US hospitals participating in the Premier Healthcare Alliance database from 2009–2011. We modeled use of CT, admission, and inpatient urologic intervention as functions of both patient characteristics and hospital characteristics.

**Results:**

Over the 2-year period, 307,612 patient visits met inclusion criteria. Among these patients, 254,211 (82.6%) had an abdominal CT scan, with 91.5% being non-contrast (“renal protocol”) CT scans. Nineteen percent of visits (58,266) resulted in admission or transfer, and 9.8% of visits (30,239) resulted in a urologic procedure as part of the index visit. On multivariable analysis male patients, Hispanic patients, uninsured patients, and privately insured patients were more likely to have a CT scan performed. Older patients and those covered by Medicare were more likely to be admitted, and once admitted, white patients and privately insured patients were more likely to have a urologic intervention. Only hospital region was associated with variation in CT rates, and this variation was minimal. Region and size of the hospital were associated with admission rates, and hospitals with more practicing urologists had higher intervention rates.

**Conclusions:**

In this dataset, the majority of patients did not require admission or immediate intervention. Despite this, the large majority received CT scans, in a cohort representing 15–20% of all US ED visits. The CT rate was minimally variable at the hospital level, but the admission rates varied 2-fold, suggesting that hospital-level factors affect patient management. The high rate of CT usage coupled with the low rate of immediate intervention suggests that further research is warranted to identify patients who are at low risk for an immediate intervention, and could potentially be managed with ultrasound alone, expectant management, or delayed CT.

## Introduction

### Background

Recent estimates suggest that there are over 2 million emergency department (ED) visits annually in the United States for suspected renal colic [1]. Despite such a high frequency of visits, controversy exists over the process of diagnosing obstructing kidney stones, as it has evolved over the past two decades [2–8]. Current evidence supports the use of both non-contrast CT and ultrasound [3,9–11]. The American College of Emergency Medicine’s “Choosing Wisely” recommendation states that clinicians should “avoid ordering CT of the abdomen and pelvis in young otherwise healthy emergency department (ED) patients (age <50) with known histories of kidney stones, or ureterolithiasis, presenting with symptoms consistent with uncomplicated renal colic,” but guidelines for the diagnosis of first-time kidney stones do not exist within the Emergency Medicine literature [12]. Urological guidelines have evolved, with 2008 guidelines unequivocally recommending CT scan [13] and more recent guidelines being less prescriptive [11]. Current guidelines may be interpreted differently by clinicians, as they state: 1. “Ultrasound (US) should be used as the primary diagnostic imaging tool,” 2. “With fever or a solitary kidney and when diagnosis is doubtful, immediate imaging is indicated,” and 3. “Following initial US assessment, non-contrast CT should be used to confirm stone diagnosis in patients with acute flank pain, because it is superior to IVU” [11]. In addition, previous studies have suggested that the performance of an inpatient intervention for kidney stones may not be driven entirely by clinical factors, and clinician availability or hospital factors may play a role [14,15].

CT scan utilization for suspected renal colic has risen dramatically in the past two decades and is now contributing to increasing concern over a national radiation burden [1,16,17]. Additionally, patients often receive more than one CT scan in the course of their acute disease process: one study demonstrated that over a period of only 10 months, 79% of patients with suspected stones received two or more CT scans [18]. Furthermore, 50% of patients with kidney stones will have another episode of renal colic within 5–10 years [19]. Coupled with increasing awareness of costs, these issues have led to an ongoing debate over the appropriate diagnostic imaging modality for ED patients with presumed first time renal colic. Ultrasound is emerging as a safe, low-cost alternative, and recently a large, pragmatic, comparative effectiveness trial demonstrated that presumed renal colic patients in an ultrasound-first pathway received less radiation without significant differences in high-risk diagnoses or serious adverse events, return emergency department visits, or hospitalizations [4]. Although some patients do go on to have CT scans, the routine use of an ultrasound-first pathway could cut CT rates in half [4].

Substituting ultrasound for CT scan in appropriate ED patients may be beneficial in regards to decreasing radiation exposure and ED costs, however, the current use of CT scans in these patients has not been quantified. While attempting to standardize the ED workup may be appropriate, no studies have examined factors that may play a role in variability. Additionally, little is known about variation in admission rates and inpatient urologic procedure rates in renal colic patients seen in the ED.

We sought to describe the state of diagnosis and management of renal colic in a diverse group of US EDs, specifically evaluating the rates of CT scanning and ultrasound use, admission and inpatient urologic procedures, and inter-institutional variability in diagnosis and management. We hypothesized that patient factors, such as age, insurance status and history of kidney stones would influence the CT, admission and procedure rates, and that controlling for patient characteristics, hospital factors would also be associated with CT scan, admission and intervention rates. Specifically, we hypothesized that hospitals with more practicing urologists would have higher intervention rates.

## Methods

### Study Design and Setting

We conducted a retrospective cohort study using claims data from hospitals that participated in the Premier database (Premier Healthcare Informatics, Charlotte, NC) between July 1, 2009 and June 30, 2011. Premier contains a date-stamped log of all items and services that the participating hospital has charged to the patient or insurer (such as medications, laboratory tests, diagnostic, and therapeutic services), in addition to the elements found in hospital claims derived from the uniform billing 04 (UB-04) form. These claims are submitted based on encounter, and ED visits are linked to inpatient admissions and procedures, but not to subsequent outpatient care. The 444 participating hospitals are geographically and structurally diverse and closely approximate the makeup of acute care hospitals nationwide. This database contains approximately 15%-20% of all US ED visits. The Premier database was created to measure healthcare utilization and quality of care and is drawn from voluntarily participating hospitals. It contains data on approximately 1 in every 4 discharges nationwide [20]. The Premier database has been used for hundreds of investigations over the past decade [20]. This study was determined to be exempt by the local Institutional Review Board.

#### Study Population

We identified subjects aged 18 or older who had an ED visit with a principal or secondary diagnosis consistent with previously described ICD-9 codes for renal colic (788.0) or kidney stone (592.0, 592.1, 592.9, 594.1, 594.2, 594.8, 594.9, 274.11) [21]. Additionally, subjects were excluded if they did not have a Clinical Classification Software [22] code consistent with kidney stone, symptoms of kidney stone, or complications of kidney stone (urinary stone, abdominal pain, UTI, sepsis, back problem, other GU diagnosis, acute renal failure, other diagnosis kidney, nausea and vomiting). For patients with multiple visits meeting inclusion criteria that fell within a three-month span, we selected the first visit, as we were attempting to capture management of a patient’s initial presentation for an episode of renal colic. As data points are coded only for month and not exact date, when two visits occurred within the same month, one was randomly dropped from the data set to avoid oversampling of repeat visits.

#### Study Protocol: Methods and Measurements/Outcomes

Outcome measures included (1) performance of a CT scan (both “renal protocol” [non-contrast helical CT] or any other CT scan of the abdomen and pelvis) (2) hospital admission (inpatient or observation status), and (3) a urologic intervention. Transfers to another hospital were grouped with admissions, presuming that patients are usually transferred for a higher level of care. In defining urologic interventions, the ten most common procedures performed in admitted renal colic patients have been previously identified but include CT, ultrasound, and transurethral clearance of the bladder (which occurred at only 1.6% of hospitalizations and is not specific to stone disease). Therefore, only the remaining 7 were counted as urologic interventions (S1 Table) [23].

### Covariates

Patient variables included age, sex, race, payer, previous ED visit or admission for renal colic (within 12 months), receipt of an ultrasound, concomitant infection/complications (pyelonephritis, sepsis, bacteremia, acute kidney failure, oliguria and anuria, acute glomerulonephritis), and comorbidities (in admitted patients only). Ultrasounds included both “complete” abdominal/kidney codes as well as “limited” procedure codes, capturing both radiology-performed ultrasound and emergency physician-performed (or “point-of-care”) ultrasounds (provided they were billed for). Concomitant infections were assessed using ICD-9-CM codes (S2 Table), and comorbidities were assessed for admitted patients using the Healthcare Cost and Utilization software [24] and the combined Charlson/Romano/Gagne comorbidities software [25].

Hospital characteristics collected included geographic region, size (beds), urban/rural location, teaching status, and number of urologists performing kidney stone procedures. Within Premier, using codes for individual physicians and specialties we compiled a count of urologists at each hospital. We then excluded any urologists who had not performed either extracorporeal shockwave lithotripsy (ESWL, ICD-9 98.51) or ureteroscopy (ICD-9 56.31) during the study period in order to exclude urologists who do not routinely perform procures for kidney stones. Analysis was restricted to hospitals that treated at least 50 eligible patients over two years in order to produce stable hospital level rate estimates.

### Data Analysis

We calculated summary statistics for patient characteristics for all included ED visits, and report the number and percent of patients seen by hospital characteristics. We assessed differences in characteristics of ED patients grouped by (1) presence of a CT scan, and (2) hospital admission/transfer, and among admitted patients grouped by performance of a urologic procedure using chi-squared tests for categorical variables and Wilcoxon rank sum tests for continuous variables (S3 Table). To assess the impact of patient characteristics on the use of non-contrast CT scan, decisions to admit, and the performance of an intervention while adjusting for other factors, we developed hierarchical generalized linear models (HGLM) for each outcome, including a random effect for hospital. Patient factors associated with an outcome with p<0.10 were included in initial models, and final models retained factors with p<0.05. Patient factors considered included age, race/ethnicity, sex, payer, receipt of an ultrasound, any prior year hospitalizations for renal colic and any prior year visits to ED for renal colic. Among admitted patients we also evaluated the impact of comorbidity burden on use of procedures. While all types of CT abdomen are reported in summary statistics, the model building only included CT scans done without PO or IV contrast (“renal colic protocol”).

To adjust for patient case mix, using the HGLM we estimated risk-standardized rates of non-contrast CT usage, hospital admission, and urological procedures. For each hospital a risk-standardized rate was estimated as the ratio of the hospital’s predicted rate (using the hospital random effect) to the hospital’s expected rate (using the average hospital effect) times the overall rate ignoring hospital. We evaluated the distributions of the risk-standardized rates across hospitals as well as correlations among the risk-standardized non-contrast CT, admission and urologic procedure rates. To assess the role of hospital characteristics we then modeled the risk-standardized hospital rates as functions of hospital characteristics via analysis of variance models.

From the HGLM we used *median odds ratios* to measure variability among hospitals. The median odds ratio is the median value obtained comparing the adjusted odds of an outcome if an individual with the same characteristics was seen at two randomly selected hospitals. As is involves comparing a higher-ranked hospital to a lower-ranked hospital, it will always be greater than or equal to one. It characterizes heterogeneity across hospitals, is adjusted for patient-level covariates, and may be directly compared against odds ratios of patient-level characteristics [26]. For example, a value of 1.50 suggests 50% higher odds of receiving a CT scan if the same patient presented at one randomly selected hospital as opposed to another [26].

Analysis was performed using SAS 9.3, STATA 13.1 (SAS Institute, Cary, NC & StataCorp. 2013. *Stata Statistical Software*: *Release 13*. College Station, TX: StataCorp LP.).

#### Missing Data

Sixty-four encounters were omitted from the original cohort for unknown sex. Missing data in the category of race or payer were included in “unknown/other” categories.

## Results

### Characteristics of Study Subjects

**Fig 1:**
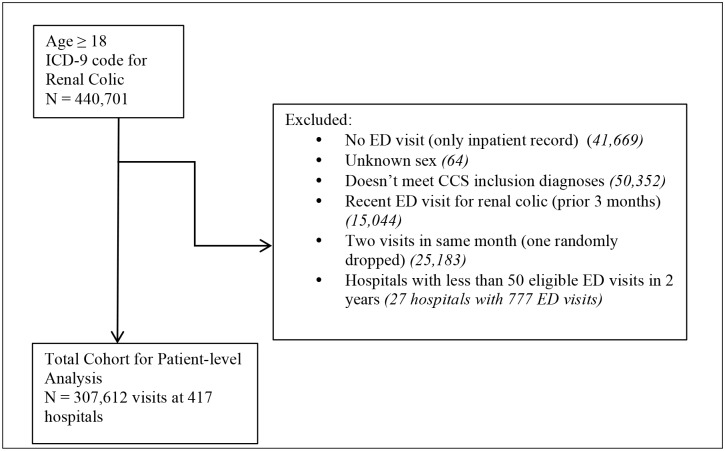
Selection of sample of patients with renal colic from Premier cohort.

Between July 1, 2009 and June 30, 2011, 440,701 adult patients met initial inclusion criteria based on ICD-9 codes, with a final study sample of 307,612 patients after applying study exclusion criteria (Fig 1). Median age was 44 years and 56.2% of patients were male (Table 1). The majority of patients included were seen at hospitals that were urban, non-teaching, and medium or large (Table 2).

**Table 1:** Characteristics of renal colic patients. (307,612 subjects).

Patient Characteristics (N = 307,612)	Overall median [IQR] or N(%)
Age	44 [33–57]
Male	172,949 (56.2%)
Race	
Black	19,537 (6.4%)
White	216,395 (70.4%)
Hispanic	20,651 (6.7%)
Other	51,029 (16.6%)
Payer	
Medicare	49,641 (16.1%)
Medicaid	31,497 (10.2%)
Private	153,085 (49.8%)
Uninsured	59,945 (19.5%)
Other/unknown	13,444 (4.4%)
Prior Visits for Renal Colic within Dataset (24 months)	
Prior ED Visit	28,387 (9.2%)
Prior Inpatient Visit	8,109 (2.6%)
Concurrent Infection/Kidney Injury	
Pyelonephritis	12,647 (4.1%)
Sepsis	8,697 (2.8%)
Acute Kidney Injury	11,231 (3.7%)
Imaging in the ED	
CT scans of Abdomen	254,211 (82.6%)
Renal Protocol	232,704 (75.6%)
Other CT Abdomen	25,993 (8.5%)
Ultrasound	18,975 (6.2%)
Hospital Admission (includes transfers)	58,266 (18.9%)
Urologic Interventions among admitted patients	29,759 (52.2%)
Urologic Interventions among all patients	30,239 (9.8%)

**Table 2:** Hospital characteristics and distribution of patients. (417 hospitals).

Hospital Characteristics (417 hospitals) and distribution of patients			
Hospital Characteristics		Number of Hospitals	Patients (%)
Setting	Rural	101	44,607 (14.5%)
	Urban	316	263,005 (85.5%)
Size by beds	Small, <200	157	71,352 (23.2%)
	Medium, 200–400	157	123,472 (40.1%)
	Large, >400	103	112,788 (36.7%)
Teaching status	No	310	215,712 (70.1%)
	Yes	107	91,900 (29.9%)
Region	Midwest	89	53,941 (17.5%)
	Northeast	58	39,035 (12.7%)
	South	184	154,769 (50.3%)
	West	86	59,867 (19.5%)

### Main Results

Of all included patients, 254,211 (82.6%) received a CT scan of their abdomen, of which 232,704 (91.5%) were renal protocol. Only 18,975 (6.2%) of the included patients had an ultrasound. The majority (81% of all patients) were discharged home from the ED, with 58,266 (18.9%) admitted to inpatient or observation status, or transferred to another hospital. Among admitted patients, 29,759 (52.2%) had an inpatient urologic intervention, which represents 9.8% of all patients diagnosed with renal colic in the ED. Of the 36,496 patients with previous ED visits for renal colic within the database (within the past 12 months and at the same hospital), the CT rate was 59% (S3 Table).

#### CT Usage

The associations between patient characteristics and CT scan rates are presented in Table 3. Female patients and those who had a recent ED or inpatient admission for renal colic were less likely to receive a CT scan. Patients receiving an ultrasound were also less likely to receive a CT scan, although nearly half (47.7%) of those who had an ultrasound also had a CT scan. Older patients, those of Hispanic ethnicity, and those with private insurance or no insurance were more likely to receive CT scans. The Median Odds Ratio for non-contrast CT scan was 1.72, meaning that individuals with the same characteristics had, at the median, a 72% higher chance of receiving a CT scan at one randomly selected hospital versus another.

**Table 3:** Adjusted associations between patient-level characteristics and CT scan use, admission, and inpatient intervention from hierarchical multivariable models.

	Odds Ratio (95% CI)		
Patient Characteristic	Renal Protocol CT Scan	Admission/Transfer	Urologic Intervention (among admitted patients)
Age (10 years increments)	1.04 (1.03–1.05)*	1.41 (1.40–1.42)*	NS
Sex			
Male	1 (ref)	1 (ref)	1 (ref)
Female	0.86 (0.85–0.88)*	1.65 (1.61–1.68)*	NS
Race			
White	1 (ref)	1 (ref)	1 (ref)
Black	0.97 (0.94–1.01)	0.97 (0.92–1.01)	0.8 (0.74–0.86)*
Hispanic	1.15 (1.1–1.2)*	0.96 (0.91–1.01)	0.93 (0.85–1.01)
Other	1.09 (1.05–1.12)*	0.90 (0.86–0.93)*	0.97 (0.91–1.04)
Insurance			
Medicare	1 (ref)	1 (ref)	1 (ref)
Medicaid	0.99 (0.95–1.03)	0.80 (0.76–0.84)*	1.09 (1.02–1.16)
Uninsured	1.1 (1.06–1.14)*	0.52 (0.50–0.54)*	1.18 (1.11–1.26)*
Private	1.21 (1.17–1.25)*	0.60 (0.58–0.62)*	1.42 (1.36–1.48)*
Prior Visit for Renal Colic in the Data Set			
Inpatient visit	0.76 (0.72–0.8)*	2.62 (2.49–2.77)*	0.86 (0.8–0.93)*
ED Visit	0.32 (0.31–0.33)*	1.15 (1.11–1.2)*	1.35 (1.26–1.44)*
Comorbidities (only available for admitted patients)			
Pyelonephritis			1.17 (1.11–1.23)
Sepsis			NS
Acute kidney Injury			1.47 (1.4–1.54)
Diabetes			0.92 (0.88–0.97)
Obesity			1.27 (1.2–1.35)
Substance abuse			0.55 (0.49–0.62)
Imaging Obtained			
Ultrasound	0.25 (0.24–0.26)	6.41 (6.18–6.65)	0.46 (0.43–0.48)
CT Scan		0.94 (0.91–0.96)	NS

* = P <0.05

NS = Not significant, not included in model

**Fig 2:**
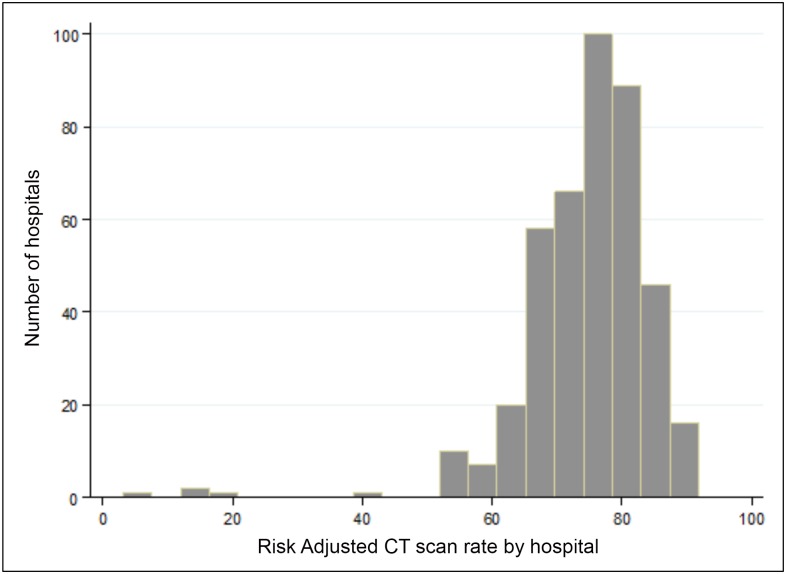
Distribution of risk-adjusted CT rates across hospitals.

There was modest variation in rates of non-contrast CT scan usage across hospitals (median = 76.4%, IQR: 69.7%-81.4%). The distribution of risk-adjusted renal protocol CT rates are shown in Table 4 and a frequency distribution of risk-adjusted CT rates is seen in Fig 2. Only region was statistically significantly associated with real CT protocol rates in multivariate analysis, with adjusted median rates ranging from 73% (West) to 78% (Midwest) (p<0.02) (Table 4).

**Table 4:** Risk-adjusted outcome rates based on the generalized linear regression model by Hospital Characteristics.

Hospital Characteristic		N	Rates (%) of Renal Protocol CT Scan median (IQR)	Rates (%) of Admission (includes transfers) median (IQR)	Rates (%) of Urologic intervention(of admitted patients) median (IQR)
All		417	76 (70–81)	14 (10–20)	55 (43–64)
Setting	Rural	101	75 (69–79)	11 (8–16)	50 (29–63)
	Urban	316	77 (71–81)	15 (11–21)	56 (47–64)
Size by beds	Small, <200	157	76 (70–81)	11 (8–16)**	49 (33–63)
	Medium, 200–400	157	77 (71–81)	15 (11–21)**	57 (48–62)
	Large, >400	103	76 (70–80)	18 (13–25)**	58 (50–66)
Teaching status	No	310	76 (70–81)	13 (9–19)	54 (41–63)
	Yes	107	77 (71–81)	17 (13–23)	56 (48–64)
Region	Midwest	89	78 (74–82)*	19 (13–23)**	58 (47–65)
	Northeast	58	73 (69–79)*	21 (15–44)**	57 (49–64)
	South	184	77 (71–81)*	12 (8–17)**	53 (42–63)
	West	86	73 (68–78)*	12 (9–16)**	52 (39–63)
Number of Urologists	1–5	89	77 (72–81)	12 (9–21)	49 (37–60)**
	6–10	97	78 (72–82)	15 (11–21)	56 (48–64)**
	11–18	97	75 (69–81)	15 (11–19)	57 (51–64)**
	19–69	88	77 (70–81)	16 (12–20)	61 (52–68)**
	Unknown	46	73 (69–78)	7 (5–12)	28 (22–41)

* p<0.02,

**p<0.0001

#### Admission Rates

Older patients and women were more likely to be admitted to the hospital (OR:1.41 [95%CI: 1.40–1.42] per decade of life and OR 1.65 [95%CI: 1.61–1.68], respectively). All insurance types were associated with a decreased likelihood of admission compared to Medicare patients. The Median Odds Ratio for admission was 6.00, meaning that an individual had a 6-fold (or 500%) higher chance of being admitted at one randomly selected hospital versus another. This indicates a large amount of variation between hospitals, regarding the admission rate for these patients.

After risk adjustment, admission rates varied by a factor of two by region: Northeast and Midwest had higher risk-adjusted admission rates than hospitals in the South or West (Table 4). Large hospitals also had nearly twice the admission rate as small hospitals (Table 4).

#### Inpatient Urologic Intervention Rates

Patients with concomitant diagnoses of pyelonephritis or acute kidney injury, but not sepsis, had increased likelihood of inpatient urological procedures (OR 1.17 for pyelonephritis and 1.47 for acute kidney injury [95%CI 1.11–1.23 and 1.4–1.54, respectively]). Black race was associated with a lower rate of intervention among admitted patients (OR 0.8 [95%CI:0.74–0.86] reference group: white race). Patients with private insurance were 42% more likely to have a urologic procedure once admitted, despite lower likelihood of admission, compared to Medicare patients (OR 1.42 [95%CI:1.36–1.48]). The Median Odds Ratio for urologic procedures was 2.02, meaning that an individual had a 2-fold increased odds of receiving a urologic intervention at one randomly selected hospital versus another. Of hospital factors examined, only number of operating urologists was associated with a higher likelihood of receiving an intervention once admitted, with a statistically significant trend observed across quartiles of urologists per hospital (p for trend <0.01).

#### Sensitivity Analysis

As our methods only included patients with a discharge or admission diagnosis consistent with renal colic, we did not include patients who were seen in the ED for *suspected* renal colic but found not to have renal colic. In order to estimate the number of additional CTs performed for this indication, we examined the symptom code for “Flank Pain” (789.0), recognizing that many patients without suspected renal colic would be included using this criteria. We found that over two years, 2,963,210 patients had a diagnosis of “Flank Pain,” and 13.46% of them were included in our study due to concurrent renal colic codes. Of the 2,564,242 who we did not include in our analysis, 16% (408,885) had non-contrast abdominal CT scans and 22%(556,330) had abdominal CTs with contrast (IV or PO), and 1% had both.

## Discussion

In this large cohort, we found that nearly 6 of 7 patients with a diagnosis of renal colic in the emergency department underwent a CT scan. In patients with previous visits within this dataset for renal colic, the CT rate was still 59%, and this group is likely a vast undercount of those with a history of renal colic. Furthermore, imaging practices at different facilities exhibited modest variation. Given increasing concern for potential harms of radiation exposure, and emerging evidence supporting the safety and utility of alternative imaging modes, these findings suggest the existence of an important population health opportunity; namely, targeted efforts to reduce unnecessary radiation exposure in the evaluation and management of renal colic.

To accomplish this goal, a critical unanswered question pertains: what clinical circumstances clearly mandate or obviate computerized tomography? Patients who need immediate urologic intervention, such as those who are septic, have only one kidney, or who are anuric, clearly warrant immediate CT scan. Conversely, the Choosing Wisely campaign recommends avoiding CT scan in healthy young patients with a history of renal colic who do not appear septic. Validation studies suggest the STONE score may help clinicians risk-stratify patients based on their likelihood of a kidney stone, and additional evidence suggests that young, healthy patients with a high likelihood of stone have a very low likelihood of dangerous alternative diagnoses [27–31]. Additionally, the risks of a radiation-induced cancer decrease as patients age, while the risks of a dangerous alternative diagnosis, such as abdominal aortic aneurysm, increase, making the risk-benefit relationship of routine CT scan different depending on the patient’s age. Despite growing evidence arguing against *routine* CT scanning, it is likely that in many clinical circumstances, some type of imaging will be necessary from the patient or clinician perspective.

If imaging is thought to be necessary, alternative, non-ionizing modalities such as ultrasound appear increasingly useful in the evaluation of suspected renal colic. An “ultrasound-first” pathway has been endorsed by several recent high-quality studies. First, a randomized controlled trial by Smith-Bindman et al. compared CT to ultrasound as initial imaging choice and found no increase in adverse outcomes in the ultrasound group and less overall radiation exposure [4]. Second, Abdel-Gawad et al. demonstrated that the addition of the “twinkling effect” may increase the ability of ultrasound to detect kidney stones to nearly the same sensitivity as non-contrast CT scan [9].

Current understanding suggests that radiation-associated cancer risks are of particular concern for younger, female patients [32]. Our results indicate that, to some extent, clinicians are somewhat less likely to utilize CT scan in these populations, although absolute rates remain very high. In contrast, individuals of Hispanic ethnicity are more likely to undergo CT scan, which is challenging to attribute to clinical reasons. While numerous studies have demonstrated racial disparities in treatment (such as the administration of pain medication), we are unaware of existing evidence suggesting that Hispanic patients receive more testing. One could speculate that language barriers may increase clinical uncertainty, contributing to higher testing rates.

Our findings confirm and extend prior research on imaging patterns for patients with suspected kidney stones. Hyams et al. demonstrated that CT rates increased in flank pain patients from 19.6% in 2000 to 45.5% in 2008 [1]. Although methodologically different in study design and data source, our results are consistent with a continuation of the trend demonstrated. Westphalen et al. examined data from 1996 to 2007 and showed a similar trend, with CT utilization rates starting at 4% and increasing to 42.5% [16]. Westphalen et al. did not include the same ICD-9 codes as we included, excluding 788.8 (“renal colic”). However, our inclusion of this code, considered a “symptom code” and less specific than ureterolithiasis codes, should only have decreased our CT scan rate. For example, when we examined the symptom code “Flank Pain” separately, we found that the CT rate was 38%, or 939,573 CT scans over 2 years, not including the 254,211 in our study cohort.

Our findings have important implications regarding radiation exposure in patients with this common medical condition. As this database represents 15–20% of all US hospitals, 254,211 CT scans (over two years) extrapolates to 637,000–850,00 CT scans performed on renal colic patients *annually* in the US, or 1.6 to 2.1 million CT scans annually, if non-contrast CT scans done in “Flank Pain” patients are included. Recent data regarding the harms of radiation suggest that depending on the age of the patient, these particular CT scans will cause between 1000 and 4200 cancers, an estimate that does not include the radiation from repeat CT scans or interventional procedures that involve imaging, such as fluoroscopy, that occur after the diagnosis of kidney stone is made [32].

Regarding the effects of hospital characteristics on variation in the outcomes examined, CT rates varied modestly, and only by region, but admission rates showed wide risk-adjusted variation, only some of which was explained by region and size of hospital. It is not clear why larger hospitals and hospitals in the Northeast and Midwest had higher admission rates: theoretically patients with more severe disease processes could self-select larger hospitals, and hospital culture by region could affect admission rates. However, the variation in inpatient urologic procedures showed some interesting trends: patients with private insurance and those with non-black race were more likely to have a procedure, once admitted, and no hospital factors affected these risk-adjusted rates other than number of operating urologists. This “availability effect” has been demonstrated before: as many stones pass spontaneously when given enough time, the immediate availability of a urologist may change procedure rates [15].

### Strengths & Limitations

This study uses established methods to capture a large and diverse set of clinical encounters. While our data cannot fully explain the trends and associations that were seen, the numbers represent the actual testing and management that occurred for over 300,000 patients.

There are multiple known limitations of using pre-existing data [33]. Claims-based data can tell us how many subjects had CT scans but cannot tell us why individual patients received the care they did. It is possible that many of these CT scans were necessary based on the clinicians’ concern for diagnoses other than renal colic. If this is the case, however, it suggests that clinicians need further tools to risk stratify patients, seen as how so many patients received a CT scan, while so few had an immediate intervention.

This study did not capture patients who were suspected of having a kidney stone but who received a symptom-based diagnosis after a negative CT scan, such as “Flank Pain,” (789.0). Therefore, this study does not reflect the management of patients with *suspected* renal colic, only those who were diagnosed with renal colic. However, recent studies have found that of patients with suspected renal colic, CTs were negative in 43% [27]. In our sensitivity analysis, we attempted to determine how many additional CTs may have been done in patients with suspected renal colic. We examined the diagnosis of “Flank Pain” and found an additional 408,885 non-contrast abdominal CT scans and 556,330 CT scans with contrast performed over the two-year period (with an overlap of about 25,000 people having both types of scans). This is consistent with previous data.

Regarding the use of ultrasound, many emergency departments use bedside (physician-performed) ultrasound without billing for it, and therefore the true rates of ultrasound are likely higher than stated. This data included both radiology-performed ultrasound and EP-performed ultrasound, but only if a claim was submitted for the procedure.

This dataset only differentiated between patients with and without a history of renal colic if the patient had been seen for renal colic at the index hospital within the past 12 months, making the 9.2% noted a drastic underestimate (Table 1). As 50% of patients have a recurrence of a kidney stone within 5–10 years, [19] assuming a mostly stable incidence of new onset of nephrolithiasis, one could expect that as many as 50% of patients in this cohort had a history of kidney stones, making the CT rate even more concerning, as the ACEP Choosing Wisely campaign suggests limiting CT scans in these patients [12].

Lastly, this database does not allow us to know who had urologic interventions immediately after discharge from the ED. If a large proportion of patients had urologic procedures within two or three days of their ED visit, it is arguable that the high rate of CT scanning is necessary. Existing evidence, however, puts the procedure rate at ~20%, including the 10% of patients who had their procedure during an inpatient stay [34,35]. Also, variations in the promptness with which an emergency physician can obtain follow-up for a patient may influence the inpatient procedure rate. For example, in areas where outpatient follow-up cannot be guaranteed due to lack of insurance, emergency physicians may theoretically admit more patients. However, one would expect to see *higher* admissions and procedure rates in areas with fewer urologists, and this was not seen.

## Conclusions

In this large cohort, over 250,000 CT scans were performed during a 2-year period, yet only 10% of the cohort underwent admission and urgent procedural intervention. As ultrasound is becoming established as a safe alternative to CT scan, future work should focus on promoting the use of ultrasound as the first-line imaging modality in young healthy patients and identifying and decreasing low-yield CT scans by identifying ways to risk-stratify patients based on the likelihood of a dangerous alternative diagnosis or the likelihood of a need for a urologic procedure. Additionally, improved risk-stratification could inform shared decision-making to engage patients in their care, potentially decreasing radiation burden and improving patient engagement.

## Supporting Information

S1 Table Procedure and Intervention Codes, as determined by current literature [23]. (DOCX)

S2 Table ICD-9 Codes identifying concomitant conditions. (DOCX)

S3 Table Univariate analysis results. (TIFF)
