# Supplementary material for: The Diagnosis and Management of Patients with Renal Colic across a Sample of US Hospitals: High CT Utilization Despite Low Rates of Admission and Inpatient Urologic Intervention
Source: PLoS One. 2017 Jan 3;12(1):e0169160. doi: 10.1371/journal.pone.0169160 (PMC5207425; doi:10.1371/journal.pone.0169160)
Supplement: S1 Table — (DOCX) [file pone.0169160.s001.docx]

**Supporting Information**

**S1 Table. Procedure and Intervention Codes, as determined by current literature [23].**

| Ureteral Catheterization | 59.8 |
| --- | --- |
| Retrograde pyelogram | 87.74 |
| Transurethral removal of obstruction from ureter and renal pelvis | 56.0 |
| Other cystoscopy | 57.32 |
| Ureteroscopy | 56.31 |
| ESWL | 98.51 |
| **CT abdomen | 88.01, 87.71 (kidney) |
| **Ultrasonography of abdomen and retroperitoneum | 88.76 |
| Percutaneous nephrostomy without fragmentation | 55.03 |
| Ultrasonic fragmentation of urinary stones | 59.95 |
| *Transurethral clearance of bladder | 57.0 |

** Imaging, not included

* Not included as not specific to ureterolithiasis
