# Supplementary material for: The Diagnosis and Management of Patients with Renal Colic across a Sample of US Hospitals: High CT Utilization Despite Low Rates of Admission and Inpatient Urologic Intervention
Source: PLoS One. 2017 Jan 3;12(1):e0169160. doi: 10.1371/journal.pone.0169160 (PMC5207425; doi:10.1371/journal.pone.0169160)
Supplement: S2 Table — (DOCX) [file pone.0169160.s002.docx]

**Supporting Information**

**S2 Table. ICD-9 Codes identifying concomitant conditions.**

| pyelonephritis unspecified | 590.80 |
| --- | --- |
| acute pyelonephritis without lesion of renal medullary necrosis | 590.10 |
| sepsis | 995.91 |
| severe sepsis | 995.92 |
| septic shock | 785.52 |
| shock unspecified | 785.50 |
| bacteremia | 790.7 |
| acute kidney failure | 584.5, 584.6, 584.7, 584.8, 584.9 |
| oliguria and anuria | 788.5 |
| Acute glomerulonephritis | 580.4, 580.0, 580.81, 580.89, 580.9 |
| Sepsis – due to specific organism | 038.0, 038.1, 038.2, 038.3, 038.4, 038.40, 038.41, 038.42, 038.43, 038.44, 038.49, 038.8, 038.9 |
