# Supplementary figures and images for: The Diagnosis and Management of Patients with Renal Colic across a Sample of US Hospitals: High CT Utilization Despite Low Rates of Admission and Inpatient Urologic Intervention
Source: PLoS One. 2017 Jan 3;12(1):e0169160. doi: 10.1371/journal.pone.0169160 (PMC5207425; doi:10.1371/journal.pone.0169160)

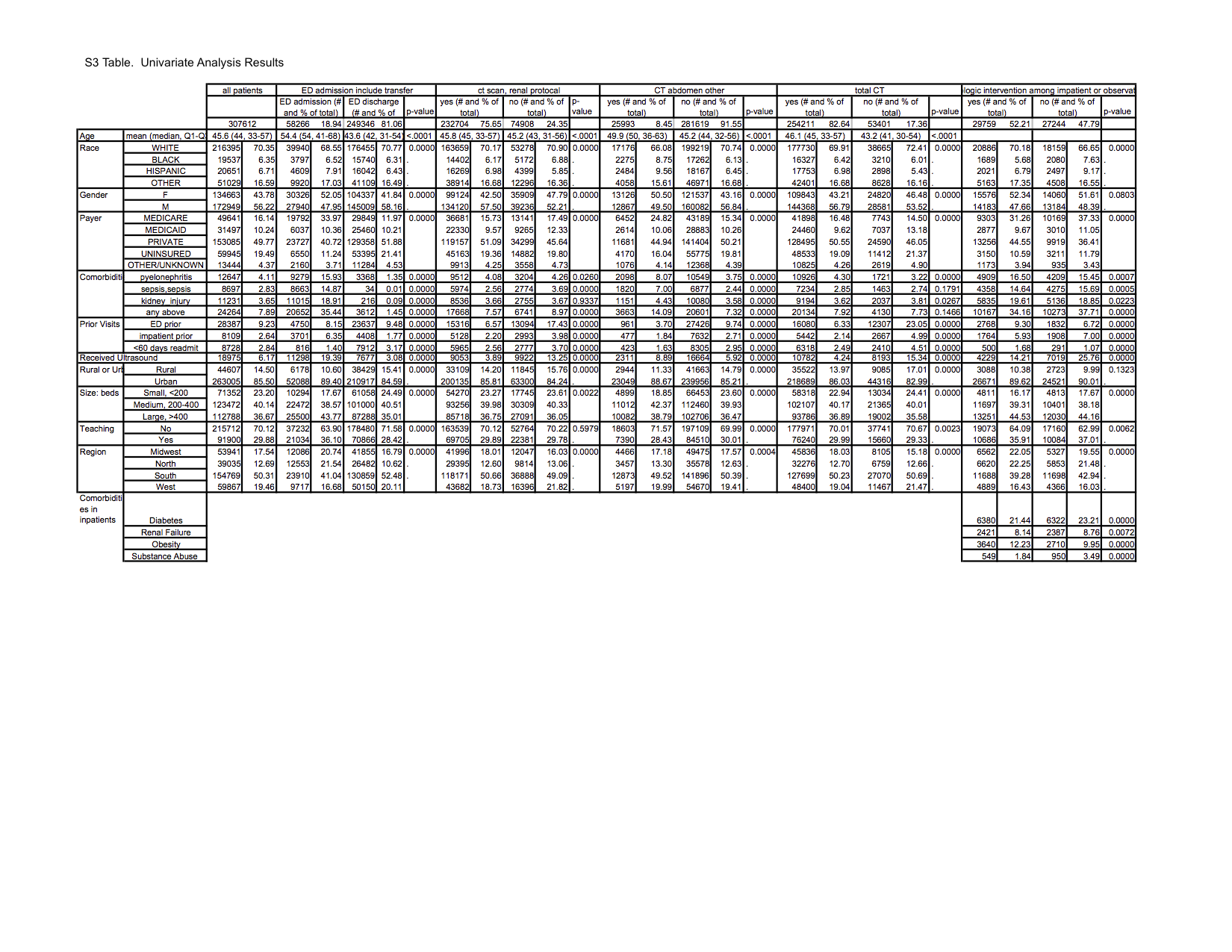

Supplement: S3 Table — (TIFF) [file pone.0169160.s003.tiff]
